# Clinical analysis of 21-gene relapse score test in hormone receptor-positive early-stage breast cancer

**DOI:** 10.3892/ol.2021.12572

**Published:** 2021-02-23

**Authors:** Lizhe Zhu, Nan Ma, Bin Wang, Can Zhou, Yu Yan, Ke Wang, Jianjun He, Yu Ren

Oncol Lett 17: 5469-5480, 2019; DOI: 10.3892/ol.2019.10277

Following the publication of the above paper, which was entitled ‘Clinical analysis of 21-gene recurrence score test in hormone receptor-positive early-stage breast cancer’, it was drawn to the Editor's attention that the use of “Recurrence Score”, as featured in the title and in the main text of the paper, was an infringement of registered trademarks held by Genomic Health, Inc. with its Oncotype DX test. It should also be noted that Genomic Health, Inc. were involved neither in the preparation of the article nor in the underlying testing with its Oncotype DX test.

A suggestion was made that the term ‘recurrence score’ could have been written as ‘relapse score’ without losing the intended meaning and without infringing upon the trademark. Therefore, the titled has been rephrased to ‘**Clinical analysis of 21-gene relapse score test in hormone receptor-positive early-stage breast cancer**’, as shown above; furthermore, ‘recurrence score’ should have been written as ‘relapse score’ at the start of the abstract on p. 5469, in the second sentence of the second paragraph of the Introduction (p. 5469), and in the titles and/or legends of Tables I–VII and Figs. 1–4.

